# Research on Compression Failure Characteristics and Damage Constitutive Model of Steel Fiber-Reinforced Concrete with 2% Copper-Coated Fibers Under Impact Load

**DOI:** 10.3390/ma17235724

**Published:** 2024-11-22

**Authors:** Guangkun Liu, Zhengxiong Bai, Wei Liu, Yajie He

**Affiliations:** 1 Yellow River Engineering Consulting Co., Ltd., Zhengzhou 450003, China; liuguangkun1990@163.com (G.L.); baizhengxiong@126.com (Z.B.); 2 State Key Laboratory of Target Vulnerability Assessment, Defense Engineering Institute, Luoyang 471000, China; 3 Key Laboratory of Water Management and Water Security for Yellow River Basin of Ministry of Water Resources, Zhengzhou 450003, China; 4 China North Industries Group Corporation Institute 53, Jinan 250031, China; 5 State Key Laboratory of Mining Response and Disaster Prevention and Control in Deep Coal Mines, Anhui University of Science and Technology, Huainan 232001, China; lll5428879@163.com

**Keywords:** steel fiber reinforced concrete, strain rate, constitutive model, failure characteristics

## Abstract

This study systematically investigates the mechanical properties of plain concrete (PC) and 2% steel fiber reinforced concrete (SFRC) under both static and dynamic loading conditions, utilizing advanced mechanical testing equipment and dynamic impact testing methods. The strain rate range studied spans from 10^−4^ s^−1^ to 483.12 s^−1^. Under static loading conditions, the maximum bearing capacity and energy absorption capacity of 2% SFRC are 2.16 times and 3.83 times greater than those of PC, respectively, indicating a significant enhancement in toughness and resistance to failure. Under dynamic loading conditions, the energy absorption capacity of SFRC increases to 6.36 times that of PC. The impact failure behavior of SFRC was analyzed using the split-Hopkinson pressure bar—digital image correlation (SHPB-DIC) method, revealing that the failure was primarily driven by splitting tension. The failure process was subsequently categorized into four distinct stages. At high strain rates, the dynamic enhancement factor, peak stress, and peak strain of SFRC exhibit a linear increase with strain rate, whereas the energy absorption capacity increases in a nonlinear manner. This study presents a simplified viscoelastic constitutive model with four parameters and develops a damage-based viscoelastic constitutive model with seven parameters, demonstrating its broad applicability. The findings offer both theoretical insights and experimental evidence to support the use of SFRC under high strain rate conditions.

## 1. Introduction

In recent years, the increasingly complex international situation, the rampant spread of terrorism, and the development and use of various types of precision-guided weapons have posed threats to many concrete structures in terms of extreme external loads such as explosions, impacts, and vibrations. For instance, on 19 April 1995, a catastrophic explosion occurred in Oklahoma City, United States. On 3 April 2017, an explosion took place in the St. Petersburg subway, Russia. On 4 August 2020, a large-scale chain explosion transpired at a seaport in northern Beirut, Lebanon. More recently, on 21 June 2023, a gas explosion occurred at a barbecue restaurant in Ningxia, China. These explosions resulted in immense destructive forces, posing significant threats to the stability of building structures and the safety of human lives and property. Research into the mechanical properties of steel fiber-reinforced concrete under impact loads is of paramount importance for advancing the study of blast resistance in such structures. Investigating the mechanical properties of concrete under impact loading is crucial for the study of blast resistance in concrete structures. As a result, this research raises higher demands on the mechanical performance of concrete [1,2,3]. Adding materials such as steel, glass, polypropylene, and carbon fibers to concrete can enhance its mechanical characteristics [4,5,6], including tensile strength, hardness, flexibility, crack resistance, fatigue durability, and impact resistance. Due to its low cost and stable performance, steel fiber-reinforced concrete is an ideal material for modification. It is a high-strength cement-based composite material widely used in highways, bridges, airport runways, coal mine tunnels, and water conservancy projects [7,8]. To address extreme external loads, increasing attention has been paid to studies on the dynamic characteristics of steel fiber-reinforced concrete.

Different types and amounts of steel fibers significantly affect the mechanical properties of concrete. Babar Ali et al. [9] demonstrated through strength tests that adding 1% steel fibers and 15% fly ash can increase the strength of recycled concrete by 13–23%. Arun Kumar Parasharh et al. [10] reported that the use of hooked-end steel fibers in concrete resulted in significant improvements in its flexural strength, compressive strength, and splitting tensile strength, with increases of 8.89%, 18.44%, and 18.80%, respectively. These results highlight the potential of using hooked-end steel fibers as a reinforcing material for concrete structures subjected to various external loads. To fabricate novel curved steel fibers, a series of experimental investigations were conducted by Jae-Jin Kim et al. [11] involving fibers with varying curvatures. The experimental findings revealed that the pull-out resistance of curved fibers was significantly superior to that of conventional S-shaped fibers, and this pull-out enhancement exhibited an increasing trend with an increase in curvature. Spelter A et al. [12] studied the long-term durability of carbon fiber reinforced concrete under environmental factors, no strength loss was found after more than 5000 h of testing, indicating that preloading improves ultimate tensile strength by more effectively aligning the filaments. Enfedaque A et al. [13] examined the impact strength of glass fiber-reinforced cementitious (GRC) panels, found that aging and glass fiber content do not significantly affect impact behavior, while numerical simulations accurately replicated the experimental results. Sabău E et al. [14] presented a novel polymer concrete composite reinforced with 30% recycled glass fibers, demonstrating homogeneous porosity distribution, lightweight properties, and a strong interface between materials, with potential for environmentally friendly construction applications. Kožar I et al. [15] presented a fiber-reinforced concrete model for capturing post-peak behavior and crack propagation, focusing on its verification through qualitative comparison with experimental results and the influence of force–displacement curve shapes. Kan W et al. [16] found that adding carbon fiber to concrete significantly improves its frost resistance, reducing mass loss and enhancing compressive strength, with optimal performance achieved at 1.5 wt.% carbon fiber content.

Extensive experimental studies by P. Mahakavi et al. [17] employed hooked-end and crimped steel fibers. The results indicated that incorporating hooked-end steel fibers led to an improvement in the compressive strength of concrete, and these fibers exhibited more favorable characteristics compared to crimped steel fibers. These investigations underscore the potential application of curved and hooked-end steel fibers as adequate reinforcements for augmenting the mechanical properties of concrete structures. Luong Pham [18] investigated the influence of different fiber lengths and volume fractions on the mechanical properties of concrete. The experimental findings showed that fiber length and volume fraction significantly affected the bonding strength of fibers in concrete. Savas and Erdem [19] investigated the effect of steel fibers and synthetic fibers with the same volume fraction on the impact resistance of IBA concrete. The results demonstrated that as the dosage of steel fibers increased, the peak stress, energy absorption value, and impact life of IBA concrete were enhanced. Sallal R. Abid et al. [20] conducted repeated impact tests on ultra-fine steel fiber reinforced self-compacting concrete at different strain rates. The results indicated that ultra-fine steel fibers could significantly improve the impact resistance of self-compacting concrete. From this perspective, current research on steel fiber-reinforced concrete mainly focuses on the influence of factors such as fiber type, fiber length, and fiber content on the mechanical properties of concrete. Relevant research on the damage and failure processes of concrete is mainly inferred through the degree of specimen damage or numerical simulation methods [21]. However, these two methods provide an indirect inference of the concrete damage and failure processes and cannot directly capture the concrete failure mechanism. Therefore, employing direct observation techniques for the analysis of the failure process is of significant importance in studying the impact failure behavior of SFRC.

Numerical simulation is a crucial research tool in the field of engineering. The accuracy of the constitutive model directly affects the precision of numerical simulation results. In contrast, the number of parameters in the constitutive model directly affects the computational efficiency of numerical simulation [22,23,24,25]. Therefore, studying constitutive models for SFRC has important guiding significance for numerical simulation. Valenzuela M et al. [26] investigated earth blocks (EB) and compressed earth blocks (CEB) made from a soil, ground recycled concrete, and water mixture, showing that CEB has significantly higher compressive and tensile strength than EB, with a validated mathematical model describing their strength and damage behavior. Chen C et al. [27] examined the mechanical properties of steel fiber reinforced concrete (SFRC) under different strain rates and fiber contents through quasi-static and dynamic tests, proposing a viscoelastic constitutive model that accurately predicts SFRC’s dynamic behavior and damage patterns. Lv Y et al. [28] investigated the mechanical properties and failure modes of fiber-reinforced high strength concrete (FRHSC) under impact using dynamic compression tests, determining strain rate effects and developing a numerical model (HJC) for simulating its behavior under large-size SHPB loading. Du et al. [29]. Proposed a super high-performance fiber-reinforced concrete constitutive model based on the HJC model, which contained 22 calculation parameters and was verified through SHPB and drop hammer impact tests. Yang et al. [30]. constructed a constitutive equation containing 15 known and eight unknown parameters, verified using LS-DYNA. Ye et al. [31]. proposed a fiber composite material compression dynamic damage constitutive model based on the HJC model and stress–strain curve under different strain rates, which is related to strain, strain rate, material strength factor, and fiber content factor. Although these constitutive models effectively characterize the mechanical properties of steel fiber-reinforced concrete, the numerous parameters involved may compromise the computational efficiency of numerical simulations. Consequently, it is essential to simplify these parameters further while maintaining accuracy.

Mohammadi et al. studied steel fiber-reinforced concrete with different volume fractions through drop hammer tests. The results showed that steel fiber-reinforced concrete with a volume fraction of 2% exhibited excellent impact resistance [32]. Therefore, steel fiber-reinforced concrete with a volume fraction of 2% is an ideal research subject. To investigate the mechanical properties and failure modes of steel fiber-reinforced concrete under impact loads, various methods such as split-Hopkinson bar, high-speed photography, and digital image correlation (DIC) were employed to conduct impact tests on steel fiber-reinforced concrete with a volume fraction of 2%. The mechanical properties and failure modes of SFRC were investigated under different strain rates, specifically at the following five strain rates: 116.25 s^−1^, 214.86 s^−1^, 380.84 s^−1^, 430.58 s^−1^, and 483.12 s^−1^. Additionally, the Zhu-Wang-Tang constitutive model was simplified based on the dynamic characteristics, and improvements were made by introducing damage factors and modification coefficients. The modified Zhu-Wang-Tang constitutive model was fitted to the experimental results, resulting in a steel fiber damage-dynamic constitutive model dependent on strain rate as the only parameter.

## 2. Experimental System and Testing Principles

### 2.1. Testing Equipment for the Experimental System

**Figure 1:**
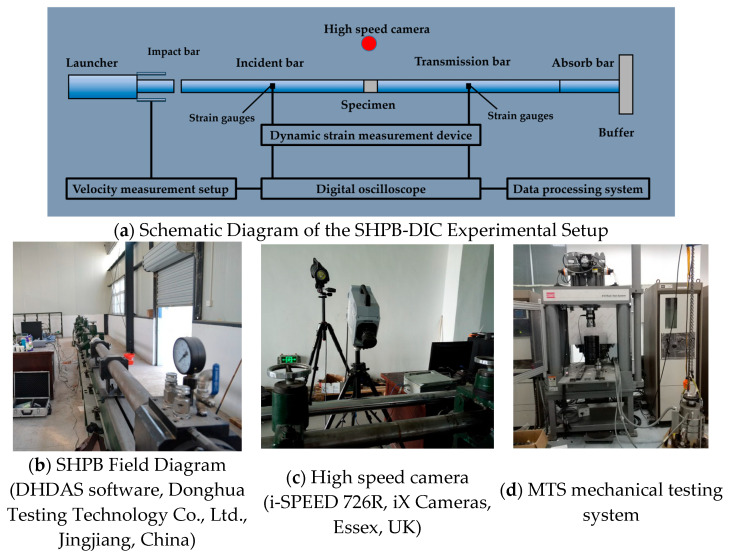
Diagram of the experimental setup.

Based on the ‘Standard For Test Methods Of Concrete Physical And Mechanical Properties’ [33], six repeated static compression tests were conducted using an Mechanical Testing & Simulation (MTS) (Eden Prairie, USA) universal testing machine. The dynamic compression experiment used a split-Hopkinson pressure bar (Diameter 75 mm, Liwei Technology Co., Ltd., Luoyang, China)—digital image correlation (SHPB-DIC), as shown in Figure 1a. The SHPB experimental system is shown in Figure 1b, which mainly consists of a gas chamber, bullet, incident bar, transmission bar, absorption bar, and absorption device. The DIC method primarily consists of a vital light source and a high-speed camera, as shown in Figure 1c. Among them, the incident bar and transmission bar are made of 60Si2Mn high-strength steel with a diameter of 75 mm, an elastic modulus of 206 GPa, and a yield strength of 1.18 GPa, which can achieve a high loading rate. This study utilizes the MTS universal mechanical testing machine to conduct static tests, as depicted in Figure 1d. The test framework exhibits an overall stiffness of 11.0 × 10^9^ N/m, with a maximum axial force capacity of 4600 kN, a maximum axial tensile output of 2300 kN, and a maximum confining pressure of 140 MPa. Additionally, the maximum pore water pressure is 140 MPa. The axial hydraulic cylinder piston has a stroke length of 100 mm. The testing space has a maximum height of 1262 mm and a minimum height of 1160 mm.

The DIC system records the entire process of specimen failure in real time. It utilizes the DIC method to capture the displacement field variations during the specimen’s failure process. Before the experiment, speckle patterns are created on the surface of the specimen. After the experiment, digital-to-analog conversion is performed to iteratively process and transform the laser speckle images at different stages. This calculation yields the variation patterns of the displacement field during different loading stages. The changes in the displacement field provide further interpretation of the specimen’s failure process. DIC is a non-contact method used to measure the surface strain field of an object. Its basic principle involves matching the variations in the speckle patterns on the object’s surface at different time points and acquiring information about the object’s surface through the motion of the speckle points.

**Figure 2:**
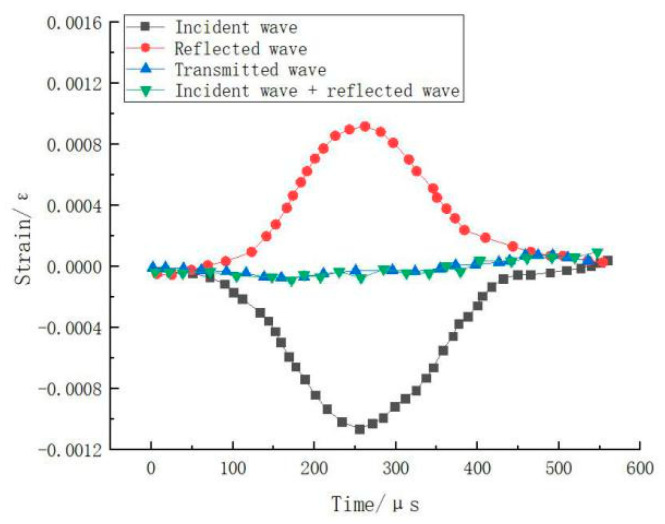
Dynamic stress balance verification.

A silicone rubber waveform shaper is affixed to the end face of the incident bar to address the issues of stress uniformity and stress balance and reduce the high-frequency oscillations of the incident wave during loading. The collected waves include the incident, reflected, and transmitted waves, as shown in Figure 2. Figure 2 shows that the transmitted wave is equal to the sum of the incident wave and reflected wave, indicating a good stress balance in this experiment. Vaseline is uniformly applied to both ends of the concrete specimen to reduce friction effects. With three repeated tests performed at each strain rate, the SHPB is used to conduct impact tests on steel fiber-reinforced concrete at five different strain rates.

The collected waves, including the incident wave, reflected wave, and transmitted wave, are shown in Figure 2. From Figure 2, it can be observed that the transmitted wave is equal to the sum of the incident wave and reflected wave, indicating a good stress balance in this experiment. Vaseline is uniformly applied to both ends of the concrete specimen to reduce friction effects. The SHPB-DIC is used to conduct impact tests on steel fiber-reinforced concrete at five different strain rates, with three repeated tests performed at each strain rate. Based on the basic assumptions of the SHPB [34,35,36], the stress, strain rate, and strain of the specimen can be calculated using the following equations via the three-wave method [37].

(1) $$\left\{\begin{array}{c} \bar{\sigma }\left(t\right)=\frac{EA}{2A_{0}}\left[\varepsilon_{i}\left(t\right)+\varepsilon_{r}\left(t\right)+\varepsilon_{t}\left(t\right)\right]\\ \bar{\dot{\varepsilon }}\left(t\right)=\frac{C}{L_{0}}\left[\varepsilon_{i}\left(t\right)-\varepsilon_{r}\left(t\right)-\varepsilon_{t}\left(t\right)\right]\\ \bar{\varepsilon }\left(t\right)=\frac{C}{L_{0}}\int_{0}^{t}\left[\varepsilon_{i}\left(t\right)-\varepsilon_{r}\left(t\right)-\varepsilon_{t}\left(t\right)\right]dt \end{array}\right.$$

In the equations, *E*, *C*_0_, and *A* represent the elastic modulus, elastic wave velocity, and cross-sectional area of the bar, respectively. $\varepsilon_{i},\varepsilon_{r},\varepsilon_{t}$ represent the strain of the incident, reflected, and transmitted waves, respectively. *A*_0_, and *L*_0_ represent the cross-sectional area and length of the specimen, respectively, while t represents the duration of the stress wave on the specimen.

By adjusting the gas pressure to control the velocity of the projectile and induce a collision with the incident rod, an elastic incident wave is generated. This wave propagates along the incident rod towards the interface of the sample. Due to the inertia of both the sample and the transmission rod, the sample undergoes compression. Given that the wave impedance of concrete is significantly lower than that of the steel rod, a portion of the incident wave is reflected back towards the incident rod, thereby becoming a reflected wave. The remainder of the incident wave is transmitted through the sample into the transmission rod, forming a transmitted wave. This transmitted wave is subsequently reflected at the free end by the absorber rod, allowing the energy within the transmitted wave to be dissipated and eventually reach a state of equilibrium.

### 2.2. Preparation of Test Specimens

**Figure 3:**
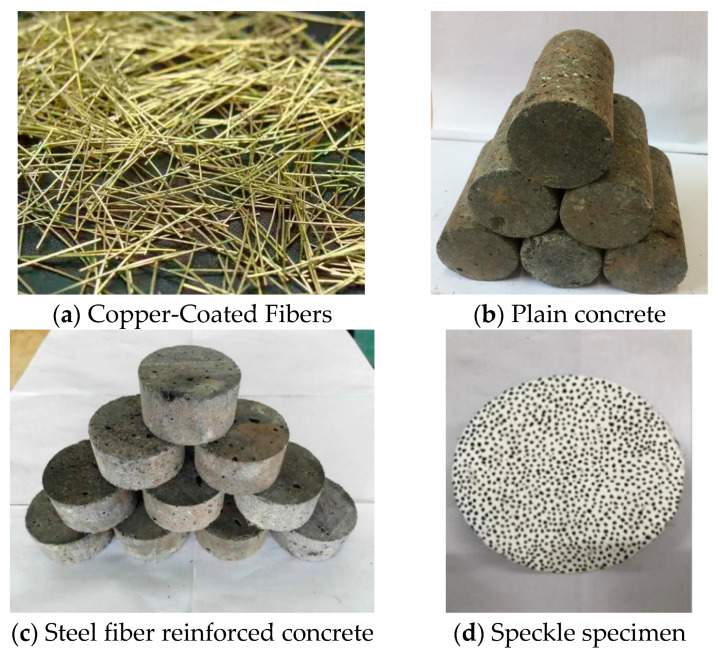
Dynamic mechanics test specimens.

This study selected steel fiber-reinforced concrete with a volume fraction of 2% as the experimental material. The C120 ultra-high-strength concrete was chosen as the base material and 6 mm ordinary crushed stones were used as the coarse aggregates. In contrast, natural river sand with a fineness modulus of 2.36 was chosen as the fine aggregate. The steel fiber-reinforced concrete was mixed with S95-grade ultrafine mineral powder, Class I fly ash, and silicon powder with a content of 95% SiO_2_. The steel fibers used were wavy in shape with dimensions of 38 mm in length, 1 mm in width, and 0.6 mm in thickness and had a tensile strength of 1000 MPa. The cement-to-sand-to-crushed stone-to-water ratio was approximately 1:1.98:2.15:0.43 [38]. The cement content was approximately 18%. The study by Mei Li et al. [39] provides some reference for specimen preparation. The steel fibers are copper-coated, with a length ranging from 5 to 15 mm, a diameter of 250 µm, and an elastic modulus of 210 GPa, as illustrated in Figure 3a. According to the testing standards established by the International Society for Rock Mechanics (ISRM), the static mechanical test specimen is a cylinder with a diameter of 50 mm and a height of 100 mm. In comparison, the dynamic mechanical test specimen is a cylinder with a height of 35 mm and a diameter of 75 mm [33]. The static mechanical test specimen is shown in Figure 3b, and the dynamic mechanical test specimen is shown in Figure 3c. To meet DIC requirements, speckles must be applied to the surface of the dynamic mechanical test specimen. To create the speckles, a layer of white paint is first applied to the surface of the specimen, followed by the application of black spots on top of the white paint. The speckled specimen is shown in Figure 3d.

## 3. Test Results and Analysis

### 3.1. Static Mechanics Experimental Analysis

**Figure 4:**
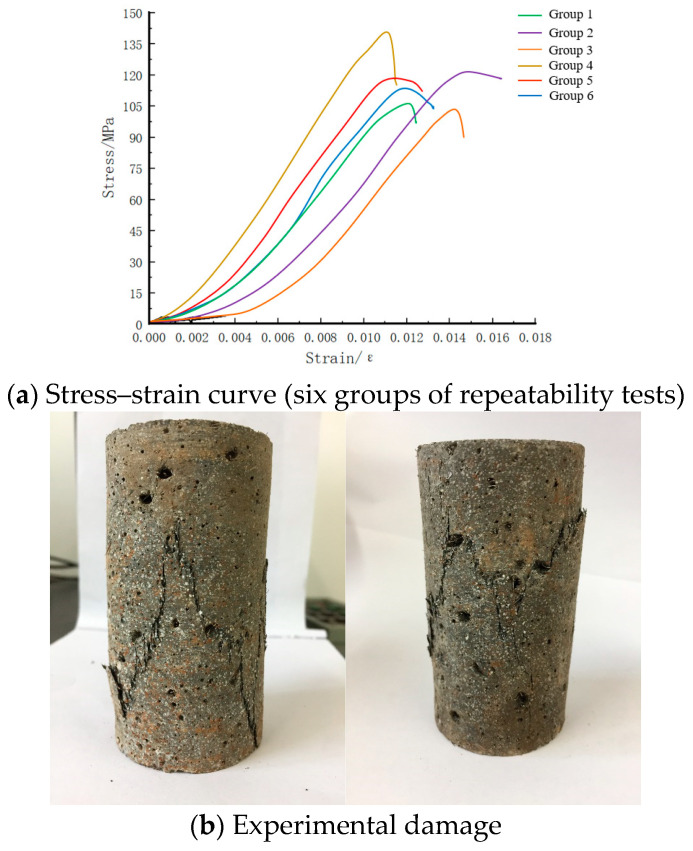
Statics experimental results.

Using an MTS mechanical testing machine, the steel fiber-reinforced concrete was subjected to six repeated static uniaxial compression tests. As shown in Figure 4a, the experimental results yielded an average peak stress of 116.67 MPa for the steel fiber-reinforced concrete. The macroscopic failure of steel fiber-reinforced concrete is shown in Figure 4b, exhibiting the characteristic of crack resistance. The steel fibers play a constraining role, delaying the initiation of new cracks and the extension of existing ones. When the length of micro-cracks exceeds the spacing between steel fibers, the steel fibers will span across the cracks to transfer the load, resulting in a more continuous and uniform stress field inside the material. This blunts the stress concentration at the tip of the micro-cracks and further constrains their propagation.

### 3.2. Analysis of Compression Failure Process and Damage Mode of Steel Fiber Concrete

**Figure 5:**
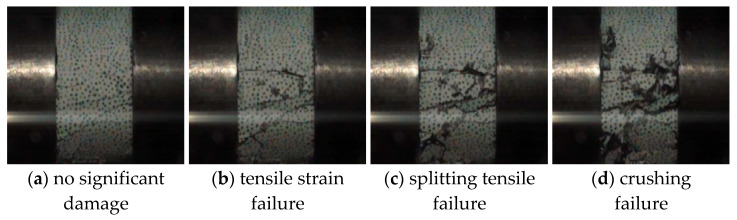
Axial impact failure process of specimen.

**Figure 6:**
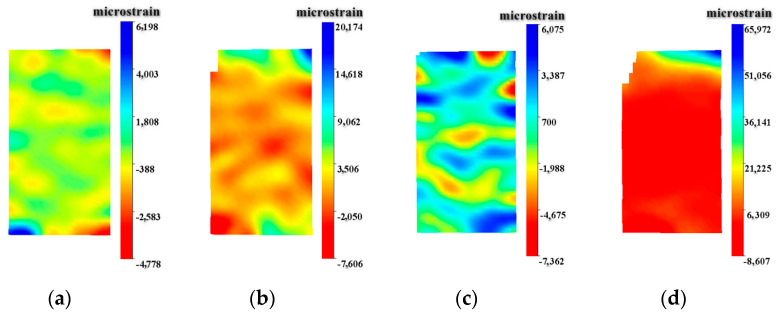
Displacement field in Y direction of specimen axial impact.

The failure process of the specimen, recorded by a high-speed camera, is shown in Figure 5. For the entire steel fiber-reinforced concrete failure process, small cracks first appear near the end close to the loading rod, accompanied by minor fragment detachment. As the loading progresses, the cracks develop and propagate along the axial direction until they penetrate the entire specimen. The displacement fields at different time intervals obtained through digital speckle correlation are depicted in Figure 6. Based on the failure states and characteristics of SFRC under impact loading observed in relevant studies [21,40,41], the failure process of SFRC depicted in Figure 5 and Figure 6 can be categorized into four distinct stages.

The first stage is the stress equilibrium stage. During this phase, stress waves reflect back and forth within the specimen until stress equilibrium is achieved. The specimen shows no significant damage at this stage, as illustrated in Figure 5a. When the incident wave reaches the surface of the specimen, the stress wave exerts a uniform force on the surface. Due to boundary effects, there is a higher effective stress at the edges of the specimen’s end face. The effective stress at the end face increases as the stress wave propagates within the specimen. Once the stress wave reaches the end face of the transmission rod, it reflects, causing the effective stress at the end face to expand from the edges inward. As the stress wave propagates, the effective stress within the specimen becomes uniform throughout. At this point, the specimen can be considered to have reached a stress equilibrium state. When the specimen achieves stress equilibrium, it satisfies the fundamental assumptions of the SHPB technique.

The second stage is the tensile strain failure stage. Once the specimen has achieved stress equilibrium, the practical stress values at various locations within the specimen continue to increase as the stress wave intensifies. Due to boundary effects, the effective stress at the circumferential boundary of the specimen is maximized, leading to failure at this location first as it reaches the allowable effective stress. This results in observable damage at the circumferential boundary, as shown in Figure 5b. Tensile strains primarily cause this failure mode; thus, it is called the tensile strain failure stage.

The third stage is the splitting tensile failure stage, as shown in Figure 5c. The tensile strains induce failure at the circumferential region of the specimen, resulting in an irregular circumferential surface. As the stress wave intensifies, this irregularity leads to an uneven distribution of effective stress internally and on the surface of the specimen. Consequently, cracks initiate axially, forming a significantly damaged axial segment. This phenomenon is attributed to axial splitting and tensile stresses; hence, it is called the splitting tensile failure stage. The predominant failure mode of the specimen is characterized by splitting tensile failure.

The fourth stage is the crushing failure stage, as shown in Figure 5d. Following the specimen’s axial splitting and tensile failure, an increase in the stress wave results in the specimen being crushed into numerous smaller fragments with reduced volume.

Figure 6 presents the strain field in the Y-direction of steel fiber ultra-high-strength concrete under impact loading. The data in Figure 6 were obtained using the digital speckle method. Negative values indicate compressive strain, while positive values represent tensile strain. In Figure 6a, the strain distribution across the entire specimen is relatively uniform during the stress equilibrium stage. Figure 6b depicts the tensile strain failure stage, where tensile strains lead to failure at the circumferential region of the specimen, resulting in a micro-strain distribution predominantly characterized by compressive strains. Figure 6c illustrates the splitting tensile failure stage, which results from both tensile and splitting tensile failure, leading to the coexistence of tensile and compressive strains with an uneven distribution. Finally, Figure 6d represents the crushing failure stage, where, under significant stress, the specimen is fragmented into small pieces, predominantly exhibiting compressive strains.

**Figure 7:**
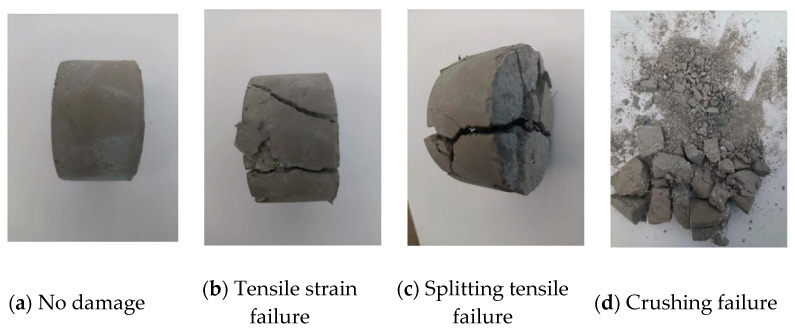
Failure mode of the specimen.

The analysis of the four failure stages shows that when the impact velocity is low, the incident stress wave reaches its peak. However, the impact intensity has not yet achieved the failure strength of the specimen, resulting in no damage to the specimen. Figure 7a shows the specimen’s undamaged state. As the impact velocity gradually increases, the stress wave has not yet peaked after completing the first stage. The specimen begins to experience tensile strain failure with the increase in stress wave intensity. When the incident stress wave reaches its peak, the specimen remains in the second stage, characterized solely by tensile strain failure, as illustrated in Figure 7b.

When the impact velocity is high, following the completion of the second stage, the incident stress wave still has not peaked. As the stress wave increases, axial splitting tensile failure begins in the specimen. At the peak of the stress wave, the specimen remains in the third stage, and the resulting damage is a combination of tensile strain failure and axial splitting tensile failure, as shown in Figure 7c.

Finally, after completing the third stage, the incident stress wave still does not reach its peak at very high impact velocities. The specimen will ultimately be fragmented into small pieces with further increases in the stress wave. The crushing failure mode is depicted in Figure 7d.

### 3.3. Dynamic Mechanical Characterization

**Figure 8:**
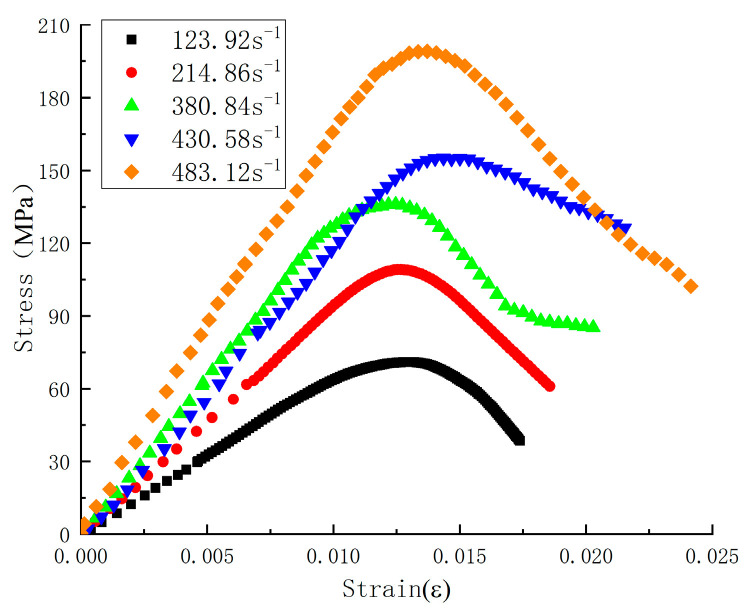
Concrete stress–strain curves at different strain rates.

Figure 8 presents the stress–strain curves of SFRC at strain rates of 123.92 s^−1^, 214.86 s^−1^, 380.84 s^−1^, 430.58 s^−1^, and 483.12 s^−1^. Table 1 summarizes the parameters of the SHPB test. The peak stress of SFRC increases with rising strain rates, indicating that this material is strain rate-sensitive. The specific reasons for the strain rate effects under impact loading are as follows:

(1) For concrete materials, the energy required to initiate internal cracks is significantly higher than that needed for crack propagation. As the strain rate increases, the number of cracks generated by the impact also increases, necessitating more incredible energy. In dynamic compression tests, the impact speed is extremely high, and the duration of the load application on the specimen is concise, leaving insufficient time for the material to absorb the shock. According to the impulse-momentum theorem, concrete can only release energy by increasing the stress path, which increases peak stress with rising strain rates.
(2) The mechanical response generated during concrete transition from a uniaxial stress to a uniaxial strain state is similar. As the strain rate increases, the lateral deformation in the center is constrained by inertial effects; the higher the strain rate, the greater the constraint. This phenomenon is akin to applying confining pressure to the concrete, increasing compressive strength with elevated strain rates.

To compare the strength of concrete under dynamic and static conditions, the Dynamic Increase Factor (DIF) is introduced, expressed by the following formula: $DIF=\frac{f_{c,d}}{f_{c,s}}$.

In the equation, $f_{c,d}$ represents the compressive strength of concrete under dynamic loading, while $f_{c,s}$ denotes the compressive strength of concrete under static loading.

**Figure 9:**
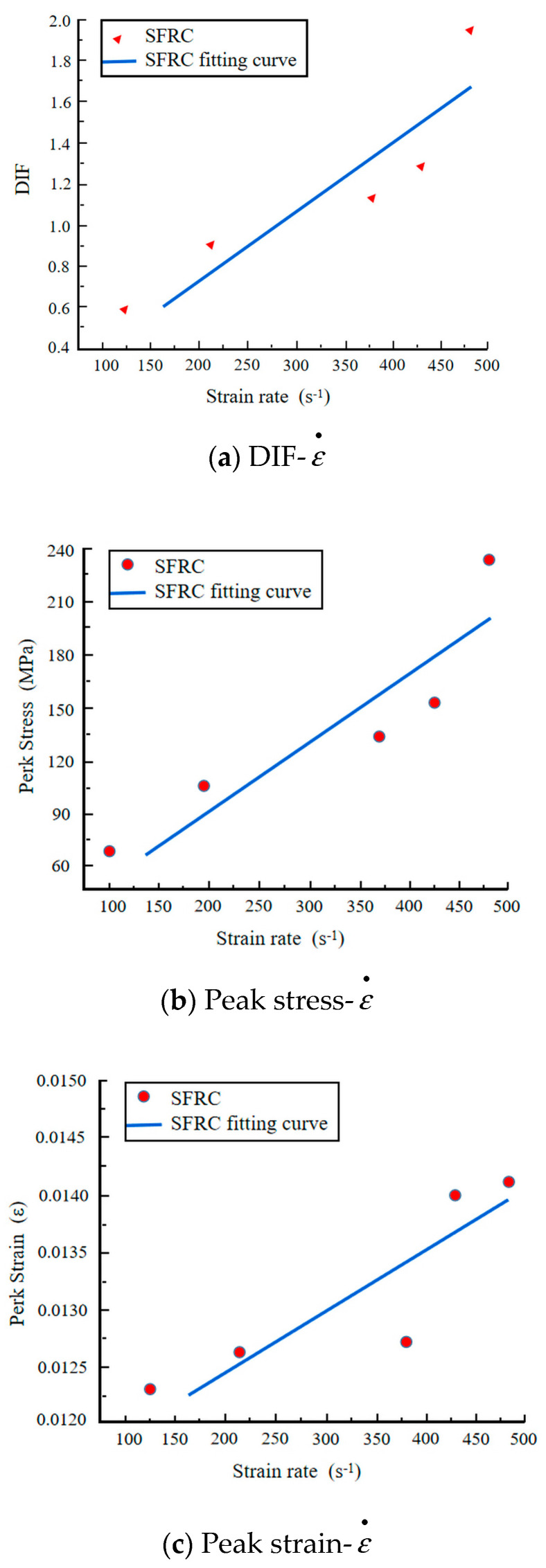
Effect of strain rate on DIF, peak stress, and peak strain.

Section 3.1 shows that the average static compressive strength of steel fiber-reinforced concrete is 116.67 MPa. The Dynamic Increase Factor (DIF) characterizes the strength variation induced by dynamic loading. As shown in Figure 9a, DIF increases with the rise in strain rate, demonstrating a clear linear relationship. Figure 9b presents the relationship between peak stress and strain rate, indicating a gradual increase in peak stress with higher strain rates, approximating a linear relationship. This further confirms the pronounced strain rate effect in steel fiber-reinforced concrete. Additionally, Figure 9c illustrates that peak strain increases linearly with the increase in strain rate.

**Figure 10:**
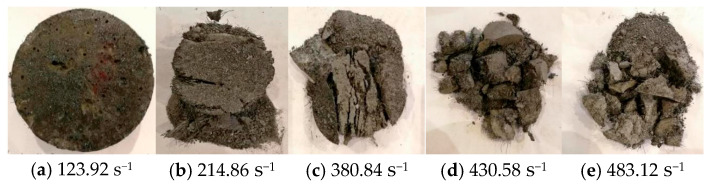
Concrete damage forms at different strain rates.

In dynamic compression tests, steel fiber-reinforced concrete exhibits non-brittle damage behavior. The failure modes of concrete at different strain rates are illustrated in Figure 10. At a strain rate of 116.25 s^−1^, no evident damage is observed in the steel fiber-reinforced concrete. At 214.86 s^−1^, significant damage occurs around the boundary of the concrete, attributed to tensile strain. At strain rates of 380.84 s^−1^ and 430.58 s^−1^, extensive fragmentation is observed, resulting from the specimen’s axial splitting and tensile failure. At 483.12 s^−1^, numerous small fragments indicate the material is in the crushing failure stage. Figure 10 demonstrates that steel fiber-reinforced concrete exhibits good toughness, with the steel fibers acting as “bridges” within the concrete matrix.

### 3.4. Analysis of Absorbed Energy of PC and Steel Fiber Concrete Under Impact Loading

Energy absorption represents the energy absorbed by the specimen during the impact process. The one-dimensional assumption of the SHPB test posits that the stress waves in the experiment conform to one-dimensional elastic conditions, where stress waves propagate along the incident and transmitted bars without attenuation. According to elastic wave theory, the calculation formula for energy absorption is as follows [42]:

(2) $$W_{I}=ACE\int_{0}^{t}\varepsilon_{i}^{2}\left(t\right)\cdot dt$$

(3) $$W_{R}=ACE\int_{0}^{t}\varepsilon_{r}^{2}\left(t\right)\cdot dt$$

(4) $$W_{T}=ACE\int_{0}^{t}\varepsilon_{t}^{2}\left(t\right)\cdot dt$$

In the equation, $W_{I}$ represents the energy of the incident wave; $W_{R}$ represents the energy of the reflected wave; $W_{T}$ represents the energy of the transmitted wave; *A* denotes the cross-sectional area of the bar; *C* indicates the velocity of elastic waves within the bar; *E* signifies the elastic modulus of the bar; $\varepsilon_{i}\left(t\right)$ corresponds to the incident wave; $\varepsilon_{r}\left(t\right)$ corresponds to the reflected wave; and $\varepsilon_{t}\left(t\right)$ corresponds to the transmitted wave.

According to the law of thermodynamic energy conservation, a portion of the total energy of the incident wave is converted into the energy of the reflected and transmitted waves. In contrast, the remaining portion is transformed into energy absorbed by the specimen’s fracturing, kinetic, thermal, and acoustic energy. The energy absorbed by the specimen’s fracturing accounts for approximately 95% of the total absorbed energy, with the sum of the remaining energies constituting about 5% of the total absorbed energy [43,44]. Due to the complexity of measuring kinetic, thermal, and acoustic energy, the energy absorbed by the specimen’s fracturing is approximated to represent the total absorbed energy, denoted as $W_{s}$. The calculation formula $W_{s}$ is as follows [42]:

(5) $$W_{s}=W_{I}-W_{R}-W_{T}$$

**Figure 11:**
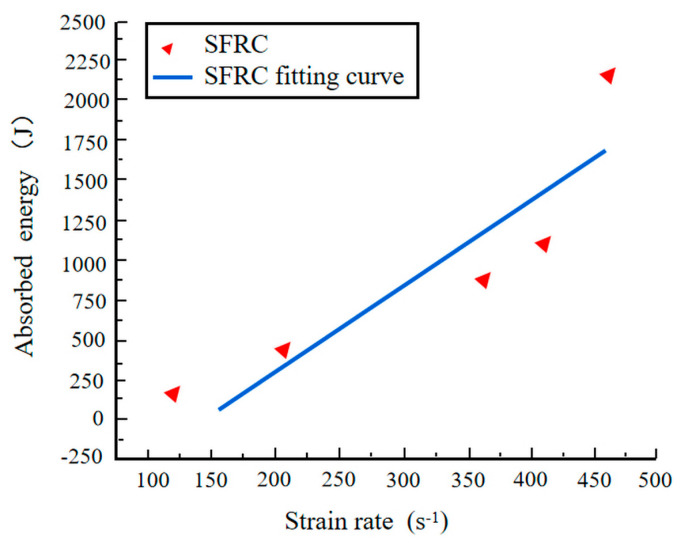
Variation curve of absorbed energy and strain rate.

Using this formula, the energy the stress wave dissipates during its propagation in the specimen can be calculated from the measured strain–time curve. Figure 11 illustrates the variation of absorbed energy with changing strain rates. PC and steel fiber-reinforced concrete exhibit increased absorbed energy with rising strain rates, demonstrating a clear linear relationship. This is attributed to the increasing number of internal cracks as the strain rate increases, with cracks transitioning from single paths to interconnected networks, resulting in numerous fracture surfaces that absorb more energy.

Define the energy absorption rate as the energy absorbed per unit strain rate, denoted as $E_{q}$, then,

(6) $$E_{q}=\frac{\Delta Q}{\Delta \dot{\varepsilon }}$$

In the equation, ΔQ represents the difference in absorbed energy, while $\Delta \dot{\varepsilon }$ denotes the difference in strain rate.

As shown in Figure 11, for PC, when the strain rate increases from 116.01 s^−1^ to 469.03 s^−1^, the absorbed energy rises from 109.76 J to 459.76 J. As the strain rate increases to 483.15 s^−1^ for steel fiber-reinforced concrete, the absorbed energy escalates from 148.21 J to 2163.04 J. For PC, for steel fiber-reinforced concrete, when characterizing the energy absorption rate of the materials using energy absorbed per unit strain rate, the energy absorption rate of steel fiber-reinforced concrete under impact loading is 6.36 times that of PC.

## 4. Ontological Modeling

**Figure 12:**
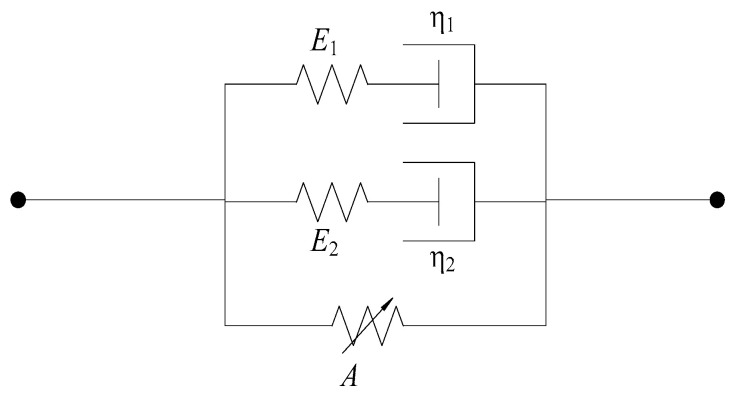
“Zhu-Wang-Tang” constitutive model.

Considering that the dynamic stress–strain curve of steel fiber-reinforced concrete exhibits significant strain hardening and strain rate hardening, the existing Zhu-Wang-Tang constitutive model effectively represents this behavior. The Zhu-Wang-Tang constitutive model is utilized to describe the material’s impact resistance, and the equation is as follows [45,46,47]:

(7) $$\begin{array}{ll} \sigma = & {\sigma_{e}+\sigma_{m1}+\sigma_{m2}=E}_{0}\varepsilon +\alpha \varepsilon^{2}+\beta \varepsilon^{3}+E_{1}\int_{0}^{t}\overset{•}{\varepsilon }\left(\tau \right)\exp\left(-\frac{t-\tau }{\theta_{1}}\right)\\ & +E_{2}\int_{0}^{t}\overset{•}{\varepsilon }\left(\tau \right)\exp\left(-\frac{t-\tau }{\theta_{2}}\right)d\tau \end{array}$$

where α, β, and $E_{0}$ represent the nonlinear spring elastic constants; the two integral terms correspond to Maxwell bodies with varying degrees of relaxation. Here, $\theta_{1}$ denotes the relaxation time of the Maxwell viscoelastic response at low strain rates, while $\theta_{2}$ indicates the relaxation time at high strain rates. $E_{1}$ and $E_{2}$ are the elastic constants of the Maxwell bodies. This constitutive model can be expressed through a mechanical model composed of spring-dashpot elements, as illustrated in Figure 12.

**Figure 13:**
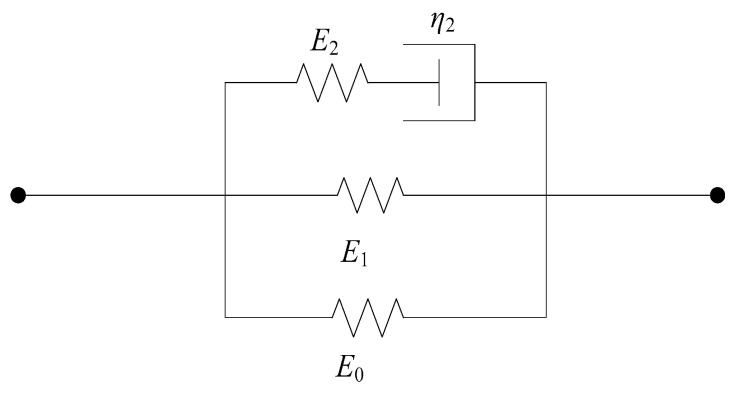
“Zhu-Wang-Tang” simplified constitutive model.

Based on the actual mechanical properties observed under dynamic loading in the experiments and the physical significance of the Zhu-Wang-Tang constitutive model, the following improvements are proposed:

(1) Regarding the first term of the equation, $\sigma_{e}=E_{0}\varepsilon +\alpha \varepsilon^{2}+\beta \varepsilon^{3}$, its significance lies in representing the strain rate-independent equilibrium stress, which characterizes the material’s nonlinear elasticity. Based on the actual deformation observed in steel fiber-reinforced concrete during experiments, where the deformation is minimal, the elastic component can be treated as linear. Consequently, the trinomial of the first term can be simplified to a monomial, that is $\sigma_{e}=E_{0}\varepsilon$, transforming the nonlinear spring in the Zhu-Wang-Tang constitutive model into a linear spring. Thus, the simplified constitutive model yields the following expression for the Zhu-Wang-Tang model:
  (8) $$\sigma =\sigma_{e}+\sigma_{m1}+\sigma_{m2}=E_{0}\varepsilon +E_{1}\int_{0}^{t}\overset{•}{\varepsilon }\left(\tau \right)\exp\left(-\frac{t-\tau }{\theta_{1}}\right)d\tau +\;E_{2}\int_{0}^{t}\overset{•}{\varepsilon }\left(\tau \right)\exp\left(-\frac{t-\tau }{\theta_{2}}\right)d\tau$$
(2) In the dynamic loading tests of steel fiber-reinforced concrete discussed in this paper, the impact loading duration is extremely brief. The loading time scale $\theta_{1}$ for the low-frequency Maxwell body ranges from 100 to 102 s. Consequently, the loading duration in this experiment is several orders of magnitude smaller than that of the low-frequency Maxwell body, which remains unrelaxed by the time the dynamic load is applied. Therefore, the low-frequency Maxwell body in the second term of the Zhu-Wang-Tang constitutive model can be represented by a simple spring. The simplified physical model is shown in Figure 13. The Zhu-Wang-Tang constitutive model equation is further simplified to:
  (9) $$\sigma =\sigma_{e}+\sigma_{m1}+\sigma_{m2}=E_{0}\varepsilon +E_{1}\varepsilon +E_{2}\int_{0}^{t}\overset{•}{\varepsilon }\left(\tau \right)\exp\left(-\frac{t-\tau }{\theta_{2}}\right)d$$

**Figure 14:**
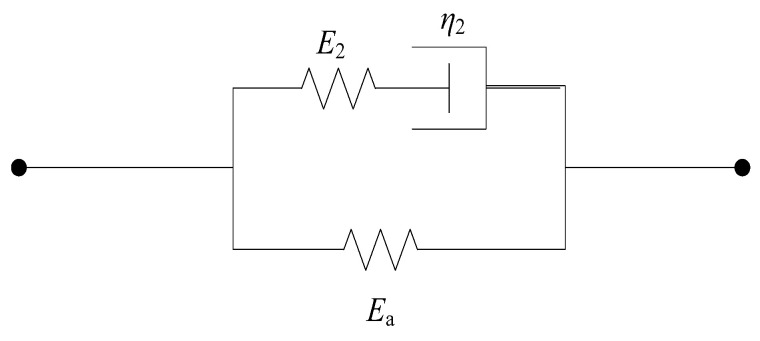
Equivalent constitutive model.

As shown in Figure 13, the springs *E*_0_ and *E*_1_ are arranged in parallel and can be replaced by a single equivalent spring $E_{a}$. The equivalent physical model is illustrated in Figure 14. The Zhu-Wang-Tang constitutive model equation is further simplified to:

(10) $$\sigma =\sigma_{e}+\sigma_{m1}+\sigma_{m2}=E_{a}\varepsilon +E_{2}\int_{0}^{t}\overset{•}{\varepsilon }\left(\tau \right)\exp\left(-\frac{t-\tau }{\theta_{2}}\right)d\tau$$

(3) During the production of composite material concrete, achieving completely uniform distribution of materials is not feasible. From a microscopic perspective, disordered distributions of micro-pores and micro-cracks exist within the material; thus, it is essential to consider damage factors when establishing the constitutive model. The distribution patterns of micro-pores and micro-cracks in concrete are highly complex and exhibit significant discreteness. A continuous damage mechanics approach is adopted to simplify the model, treating composite material concrete as a constant medium. By introducing a macro damage variable (*D*) to assess the damage extent, the primary form of the constitutive model is derived based on Lemaitre’s strain equivalence principle as follows:

(11) $$\sigma_{a}=\left(1-D\right)\sigma_{r}$$

In the equation, $\sigma_{a}$ represents the effective stress; $\sigma_{r}$ denotes the original stress; and *D* is the damage variable.

Both strain and strain rate significantly influence the damage evolution process, and the form of the damage evolution equation is as follows:

(12) $$D=A\times {\left(\frac{\overset{•}{\varepsilon }}{\overset{•}{\varepsilon_{0}}}\right)}^{\alpha }\varepsilon^{\beta }$$

In the equation, ${\overset{•}{\varepsilon }}_{0}=10^{-4}s^{-1}$ denotes the reference value of the strain rate; $\overset{•}{\varepsilon }$ represents the strain rate; A, α and β are undetermined coefficients.

In summary, combining Formulas (10)–(12) yields:

(13) $$\sigma_{a}=\left(1-D\right)\sigma_{r}=\left[1-A{\left(\frac{\overset{•}{\varepsilon }}{\overset{•}{\varepsilon_{0}}}\right)}^{\alpha }\varepsilon^{\beta }\right]\left[E_{a}\varepsilon +E_{2}\int_{0}^{t}\overset{•}{\varepsilon }\left(\tau \right)\exp\left(-\frac{t-\tau }{\theta_{2}}\right)d\tau \right]$$

Considering that the SHPB test approximates a constant strain rate, the constitutive model can be rewritten as:

(14) σa=1−Dσr=1−Aε•ε0•αεβEaε+E2θ2ε•1−exp⁡−εθ2ε•

Although the improved Zhu-Wang-Tang constitutive model presented in this paper considers damage factors, it still assumes the material is continuous. In reality, steel fiber-reinforced concrete exhibits a range of uncertainties, such as voids, cracks, and inhomogeneities, which may cause the stress peak in the stress–strain curve to occur earlier or later than expected. A correction factor δ is introduced to adjust the fitted curve to characterize these uncertainties. The final dynamic damage constitutive model, incorporating the correction factor, is expressed as follows:

(15) σa=1−Dσr=1−Aε•ε0•αε+δβ{Eaε+δ+E2θ2ε•1−exp⁡−ε+δθ2ε•}

## 5. Application of Dynamic Damage Ontology Modeling in Experimentation

### Application of Dynamic Damage Principal Model to Steel Fiber Concrete Testing

**Figure 15:**
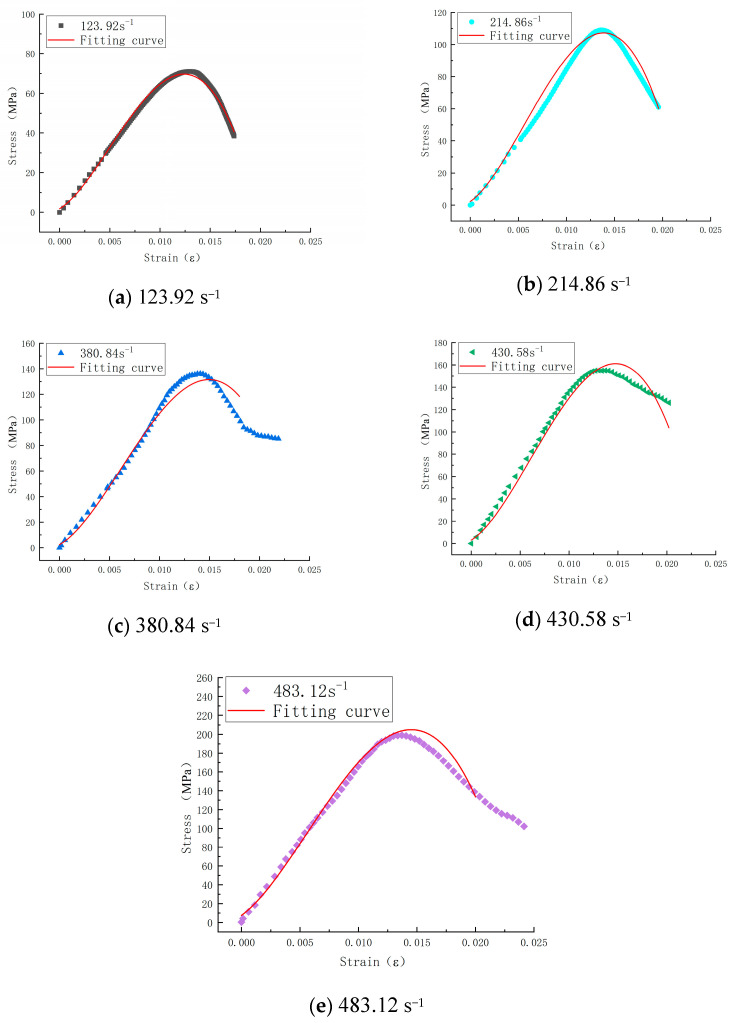
Effect of steel fiber concrete fitting curve.

In this study, Equation (15) is used to fit the stress–strain curves of steel fiber-reinforced concrete within the strain rate range of 123.92 s^−1^ to 483.12 s^−1^. The resulting fitted curve is illustrated in Figure 15, with the fitting parameters provided in Table 2. The fitted curve shows a good correspondence with the experimental data, indicating that the constructed constitutive model effectively captures the dynamic damage constitutive relationship of steel fiber-reinforced concrete.

**Figure 16:**
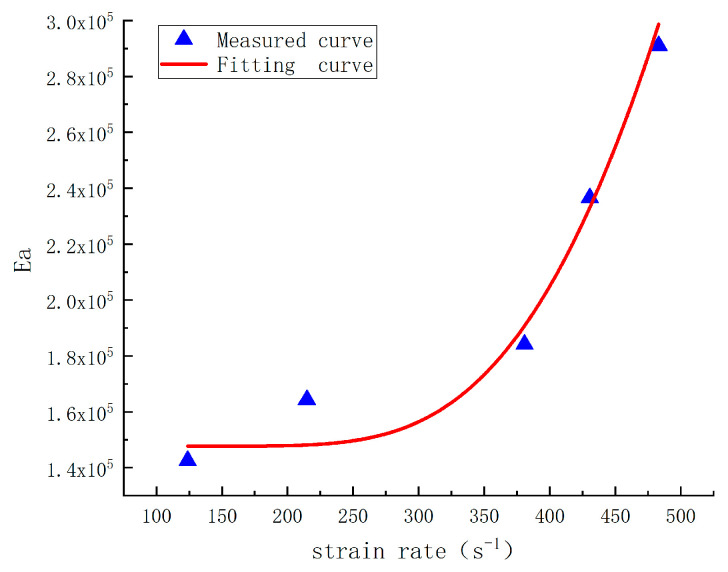
Relationship curve of *E_a_* with strain rate increase.

Parameter Analysis:

(1) Linear Term Coefficient *E_a_*
  The relationship between the elastic constant *E_a_* and strain rate $\overset{•}{\varepsilon }$ is shown in Figure 16, represented as:
  (16) $$E_{a}=A\times e^{B/x}+C$$
  where *A* = 1.61e^7;^ *B* = −2556; *C* = 147702.
(2) Elastic Constant *E*_2_

The negative value of *E*_2_ indicates structural instability of the material at high strain rates, which aligns with the physical significance of *E*_2_ as the elastic constant of a high-frequency Maxwell body. According to Table 2, the values of *E_a_* and *E*_2_ are approximately opposites; thus, *E*_2_ can be substituted with $E_{a}$, further simplifying the constitutive model as follows:

(17) σa=1−Dσr=1−Aε•ε0•αε+δβEa{ε+δ−θ2ε•1−exp⁡−ε+δθ2ε•}

Substituting Equation (16) into Equation (17) yields:

(18) σa=1−Dσr=1−Aε•ε0•αε+δβA∗eB/x+Cε+δ−θ2ε•1−exp⁡−ε+δθ2ε•

where *A* = 3000; *α* = 1; *β* = 3; ${\overset{•}{\varepsilon }}_{0}=10^{-4}s^{-1}{;\;\theta }_{2}=3;\delta$ = 0.0015.

By substituting specific values into Equation (18), we obtain the expression for the dynamic damage constitutive model of steel fiber-reinforced concrete, which is dependent on a single parameter related to the strain rate $\overset{•}{\varepsilon }$:

(19) $$\sigma_{a}=\left(1-D\right)\sigma_{r}=\left[1-3000{{\left(\frac{\overset{•}{\varepsilon }}{10^{-4}}\right)}^{1}\left(\varepsilon +0.0011\right)}^{3}\right]*\left(1.61e7*e^{-2256/\varepsilon }+147702\right)\;*\left\{\left(\varepsilon +0.0015\right)-3*\overset{•}{\varepsilon }\left[1-\exp\left(-\frac{\left(\varepsilon +0.001\right)}{3*\overset{•}{\varepsilon }}\right)\right]\right\}$$

The fitted curve demonstrates a good correspondence with the experimental curve, indicating that the constructed dynamic damage constitutive model effectively reflects the dynamic damage constitutive relationship of steel fiber-reinforced concrete. Within the strain rate range of 123.92 s^−1^ to 483.12 s^−1^, the provided dynamic damage constitutive model can directly yield the stress–strain curves corresponding to these strain rates as a reference for future research on steel fiber-reinforced concrete.

This study utilizes the SHPB to conduct dynamic tests at various loading rates, examining the failure process and failure modes of SFRC. However, several limitations exist within the scope of this research, including constraints related to both test costs and time. The experiments focused on concrete specimens with a steel fiber content of 2%, and due to limitations of the testing equipment, the strain rate range in this study was confined to 123.92 s^−1^ to 483.12 s^−1^. Building upon the findings of this study, future research could explore a broader range of fiber contents and extend the strain rate range to gain further insights into the material behavior under dynamic loading.

## 6. Conclusions

Using the SHPB-DIC method, impact tests were conducted on steel fiber-reinforced concrete with a volume fraction of 2% at different strain rates. The study investigated the impact failure process and failure modes of steel fiber-reinforced concrete and established a dynamic damage viscoelastic constitutive model specifically for this material. The main conclusions are as follows:

(1) Under static loading conditions, SFRC demonstrates a marked improvement in performance relative to PC. Specifically, the maximum bearing capacity of SFRC is 2.16 times greater, and its energy absorption capacity is 3.83 times higher than that of PC, highlighting its superior toughness and resistance to damage. Under dynamic loading conditions, both materials exhibit strain rate sensitivity; however, the energy absorption capacity of SFRC is further enhanced, reaching 6.36 times that of PC. These results underscore the exceptional performance of SFRC in high strain rate environments.
(2) The failure process of SFRC at various strain rates was investigated through impact tests employing the SHPB combined with DIC techniques. The results indicate that the failure of SFRC is primarily driven by splitting tensile failure. The failure process can be categorized into four distinct stages: the stress equilibrium stage, the tensile strain failure stage, the splitting tensile failure stage, and the crushing failure stage. These findings provide a crucial foundation for understanding the damage mechanisms of SFRC under dynamic loading conditions.
(3) In the high strain rate range (123.92 s^−1^ to 483.12 s^−1^), the dynamic enhancement factor (DIF), peak stress, and peak strain of SFRC exhibit a linear relationship with the strain rate, while its energy absorption capacity demonstrates nonlinear growth. These results further corroborate the superior energy absorption and impact resistance of SFRC under high strain rate conditions, highlighting its suitability for applications that demand high dynamic performance.
(4) Based on the impact dynamic characteristics of SFRC, a simplified dynamic viscoelastic constitutive model with four parameters is proposed. This model effectively captures the dynamic mechanical behavior of SFRC while significantly enhancing computational efficiency. Additionally, a damage-based viscoelastic constitutive model, incorporating seven parameters, was developed by integrating damage factors and correction coefficients. The model’s broad applicability was validated through experimental data. This simplified constitutive model not only facilitates generalization, but also provides a practical framework for future research to derive stress–strain relationships at specific strain rates.

## Figures and Tables

**Table 1:** Summary of Dynamic Test Data.

Number	Strain Rate (s^−1^)	Peak Stress (MPa)	Peak Strain (10^−2^)
1	123.92	80.87	1.23
2	214.86	108.21	1.26
3	380.84	135.11	1.27
4	430.58	154.61	1.40
5	483.12	233.66	1.41

**Table 2:** Fitting parameters of SFRC at different strain rates.

Strain Rate (s^−1^)	*A*	*α*	*β*	$E_{a}$	*E*_2_	$\theta_{2}$	δ
123.92	3	0.9	0.8	142,519.37	−142,523.36	3	0.001
214.86	3	0.9	0.8	164,298.04	−164,300.99	3	0.001
380.84	3	0.9	0.8	184,109.75	−184,111.75	3	0.001
430.58	3	0.9	0.8	236,492.57	−236,494.82	3	0.001
483.12	3	0.9	0.8	290,941.01	−290,943.51	3	0.001

## Data Availability

The original contributions presented in this study are included in the article. Further inquiries can be directed to the corresponding author.
